# Improving the Communication and Credibility of Government Media in Response to Public Health Emergencies: Analysis of Tweets From the WeChat Official Accounts of 10 Chinese Health Commissioners

**DOI:** 10.3389/fpubh.2022.900776

**Published:** 2022-07-22

**Authors:** Zhigang Li, Manjia Wang, Jialong Zhong, Yiling Ren

**Affiliations:** College of Management, Chengdu University of Technology, Chengdu, China

**Keywords:** public health emergency, official WeChat account, government media, informetrics, topic mining

## Abstract

**Background:**

A significant public health emergency has appeared worldwide since the beginning of 2020. The spread of negative information about COVID-19 on social media poses a challenge and threat to public health disposition and the credibility of government public opinion.

**Objective:**

This study aimed to analyze the rules and characteristics of government media in disseminating information on public emergencies. In addition, find ways and means to improve government media's communication power and credibility.

**Method:**

Based on relevant theories and measures of information econometrics, 10 WeChat official accounts of the Chinese government were taken as examples. The Python crawler tool was used to collect data of 10 WeChat official accounts-related tweets. In addition, this study used various tools, such as ROST, UCINET, and SPSS, for statistical analysis and co-word analysis of the data.

**Result:**

From January 17 to March 31, 2020, 6,612 COVID-19-related tweets were published by 10 WeChat official accounts, which broadcast epidemic overview, epidemic prevention and control, science and disinformation, epidemic assistance, epidemic impact, and negative impact. By analyzing the posting time and content of the tweets, we found that changes in the number of articles posted by the WeChat and changes in content and the progress of the COVID-19 pandemic are nearly synchronized, and most tweets are published at 8:00 am. Furthermore, based on the analytics of high dissemination index and high-frequency words, we propose that there is a significant correlation between the strength of independence and the credibility of the WeChat official account.

**Conclusion:**

The three elements of WeChat communication (value, interest, and moving) and the degree of independent innovation of public numbers impact the communication power and credibility of government media. First, if the articles published by the WeChat official account are valuable, interesting, and moving, the communication power of the WeChat official account would get more powerful. Second, increased ability for independent innovation has a positive impact on enhancing the WeChat official account's credibility. Third, government media can improve its governance effects of public health emergencies by enhancing their communication power and credibility.

## Introduction

At the beginning of 2020, the COVID-19 epidemic broke out in Wuhan, Hubei, and quickly spread worldwide, gradually evolving into a global pandemic (1, 2). Furthermore, since the advent of Web 2.0 technology, social media has become the leading platform for the public dissemination and acquisition of information. Therefore, the “infodemic” (a combination of information and epidemic) spawned by the COVID-19 pandemic is likewise sweeping the globe uncontrolled (3). In this context, the public requires comprehensive and accurate information about COVID-19-related prevention and control strategies and the number of infections *via* social media to assist individuals in taking the necessary precautions (4). Therefore, the Chinese government requires active promotion of prevention and control and health education through mass media, online media, slogans, and banners.

In China, WeChat is the most dynamic and vital social platform (5), with more than 1.1 billion monthly active users (6). WeChat releases information quickly and conveniently. These official accounts are essential for disseminating public health information (7, 8). Government media serves as a bridge for public communication. The government disseminates information to influence public opinion and improve the government's reputation (9). When a public health incident happens, improving the information governance and dissemination capacities of the WeChat official account can help enhance public opinion governance.

In handling major public health emergencies, social media is becoming increasingly important. In the complex and changing network, the data behaviors of users on different social platforms are also very different. Managing user data in different network projects through crisis response models can improve management efficiency and reduce the number of crises (10). The study of government media platforms is helpful for public opinion crisis management of public health emergencies. Government social media platforms can be used to understand the themes and hot topics of public health emergencies. By analyzing the Sina Weibo of People's Daily, which represents the official government, based on rooting theory, we can clarify the public attention and understand the public participation behavior to improve the crisis management ability (11). During the COVID-19 pandemic, one researcher used Twitter tweets to examine 10 emerging national themes to highlight governance needs (12). State-owned media can also speak out on behalf of the government and analyze state-owned media's Sina Weibo posts to build a method for averting public health problems (13). We can enhance the effectiveness of government-population communication and regulation of public health events by analyzing Chinese government social media posts during the COVID-19 pandemic to prevent public blame (14). Governments can use social media to affect public behavior in various ways. Analyzing the emotional value of posts made on the Health China Weibo, one research discovered techniques to encourage public engagement in discussions of crisis occurrences and minimize the perception of public crisis (15). To influence public opinion, the government employs a knowledge Q&A community to track changes in user sentiment, establish community communication networks, and manage opinion leaders (16). Predicting Twitter user involvement through social media posts and communication techniques may also aid pandemic management (17). A study of government social media reveals why the public participates in government media discussions (18). The government may utilize media channels to enhance user information security awareness (19). Users' risk perceptions are influenced positively by government media (20). Government media may improve public governance and make public conduct more controllable (21). By upgrading the disclosure method for personal outbreak information and increasing the rate of information sharing among internet users, the government has increased the efficiency of COVID-19 prevention and control (22).

Due to the advances in multimedia technologies, the progression of the disease may now be evaluated and anticipated in phases through official media. In the early phases of the pandemic, the government used Weibo post data to determine the outbreak's stage of development, allowing them to take proper steps (23). According to an analysis of Chinese Weibo user postings, it can be found that the method for debunking rumors varies based on the type of rumor (24). In Macau, different levels of information disclosure to varying epidemic stages through the government's Facebook have effectively addressed the public health crisis (25). Also, the government's different representations on social media can impact the development of the epidemic, and the government needs to build the trust of the population at the beginning of the epidemic (26). During a public health emergency, people can find a sense of belonging and reduce their anxiety by using social media, podcasts, websites, and other platforms (27). The government's use of social media can enhance digital health literacy in preparation for a future pandemic (28). Meanwhile, people prefer warm social media to cold e-government (29). Of course, government social media disclosure of information during an epidemic is also essential, as shown by the case study of Portugal, where information disclosure reduced the health crisis of the population (30). Network information security has a vital role in people's lives and health, and the current analysis of a variety of network traffic indicators can effectively monitor the emergence of crises to take action in advance (31). Therefore, traffic information analysis of the WeChat official account is also an effective initiative for the public health information security crisis. However, there is a lack of research on how the WeChat official account responds to major public emergencies. There is also a shortage of studies on how governments may improve public trust and involvement by strengthening their communication power and credibility to reduce public health crisis fear. The government should use public opinion control to advise the people on the proper treatment of public health emergencies. In addition, in the era of globalization with the development of the Internet, how the government media in each country can enhance their communication power and credibility during public health emergencies is also an urgent issue.

Therefore, this study aims to analyze the information characteristics of WeChat official account tweets under major public health emergencies. The information related to 10 WeChat official accounts during the epidemic was studied through statistical analysis, content analysis, and social network analysis. This study creates a foundation for increasing public health emergency information transmission and reliability *via* government channels like WeChat. Furthermore, this study makes valuable recommendations for the government to better create and convey public policy in the future when dealing with public health incidents.

## Materials and Methods

### Data Source

The Government Health and Wellness Committee as the manager of national health affairs and the authoritative information publisher can act in a timely fashion in the face of public health emergencies, releasing relevant information accurately and comprehensively. The source of this information is comprehensive and reliable and can fully show the development process of the novel coronavirus. Based on the early and late occurrence of the epidemic in each province and city, the size of the event, and the development status of the town, 10 representative WeChat official accounts of the Health and Wellness Commission were selected for this study, namely, Healthy Beijing, Healthy Guangdong, Healthy Hubei, Healthy Hunan, Healthy Jiangsu, Healthy Shanghai, Healthy Sichuan Official, Healthy Tianjin, Healthy Zhejiang, and Chongqing Health and Hygiene.

This investigation correlates with the COVID-19 epidemic and employs a Python crawler program to collect content from 10 WeChat official accounts. The data for all articles published between January 17 and March 31, 2020 were gathered from 10 WeChat official accounts. It includes nine attributes: title, originality, reading quantity, posting time, number of watches, number of likes, number of comments, article link, and article cover link. For the topic of this study, the data were manually pre-processed to remove the data of tweets not related to this public health emergency, and the final number of 6,612 tweets from 10 WeChat official accounts was obtained (Table 1).

**Table 1:** Number of public media tweets from the Government Health and Wellness Committee.

**Public nickname**	**Number of tweets**
Healthy Beijing	242
Healthy Guangdong	1,174
Healthy Hubei	904
Healthy Hunan	70
Healthy Jiangsu	475
Healthy Shanghai	360
Healthy Sichuan official	771
Healthy Tianjin	1,586
Healthy Zhejiang	543
Chongqing Health and hygiene	487
Total	6,612

### Data Processing

Information release types of public health emergencies are generally divided into four categories: public emergency notification, event science, prevention and control dynamics, and media news (32). In conjunction with this study, the content of the tweets about public health emergencies from the WeChat official account of the government media Health and Wellness Commission was further analyzed. The COVID-19 pandemic public health emergency tweets were divided into six categories based on the peculiarities of the current environment (Table 2).

**Table 2:** Classification of the type of public health emergency tweets, number, and quantity.

**Number**	**Type**	**Content covered**	**Quantity**
1	Epidemic overview release	Briefing data/leadership search	1,210
2	Epidemic prevention and control measures	Government policy/leadership voice/press conference	1,362
3	Science and disinformation	Epidemic prevention science/general health knowledge/rumor disinformation	1,739
4	Epidemic line assistance stories	Volunteer assistance/volunteer deeds/epidemic warming stories	1,952
5	Epidemic impact	Resumption of work and school/unsealing the city	275
6	Negative impact	Corruption and deception/concealment of illness	74

The data collection also yielded the number of reads and in-views for the 10 WeChat official accounts. The number of readers for each article indicates how many people have read the article; if the maximum number of readers is beyond 100,000, the particular number will no longer be displayed. The number of likes for each article indicates that the general public has read it, agreed with and enjoyed the content, and shared it with their friends. Since the number of reports of the 10 WeChat official accounts is different, the average reading of each WeChat official account is averaged for this reason, and its average value is 9,680 reads. Therefore, the average rate of reads over 10,000 times and the average number of likes were chosen as the measure in this study for observation (Table 3).

**Table 3:** Reading in the number of the statistics table.

**Public nickname**	**Rate of reads**	**Average number**
	**over 10,000 times**	**of likes**
Healthy Beijing	0.29	29.34
Healthy Guangdong	0.20	70.85
Healthy Hubei	0.86	173.87
Healthy Hunan	0.00	3.91
Healthy Jiangsu	0.16	33.38
Healthy Shanghai	0.29	43.59
Healthy Sichuan official	0.25	47.66
Healthy Tianjin	0.09	16.76
Healthy Zhejiang	0.32	126.84
Chongqing Health and hygiene	0.02	5.37

### Research Methods and Tools

**Figure 1:**
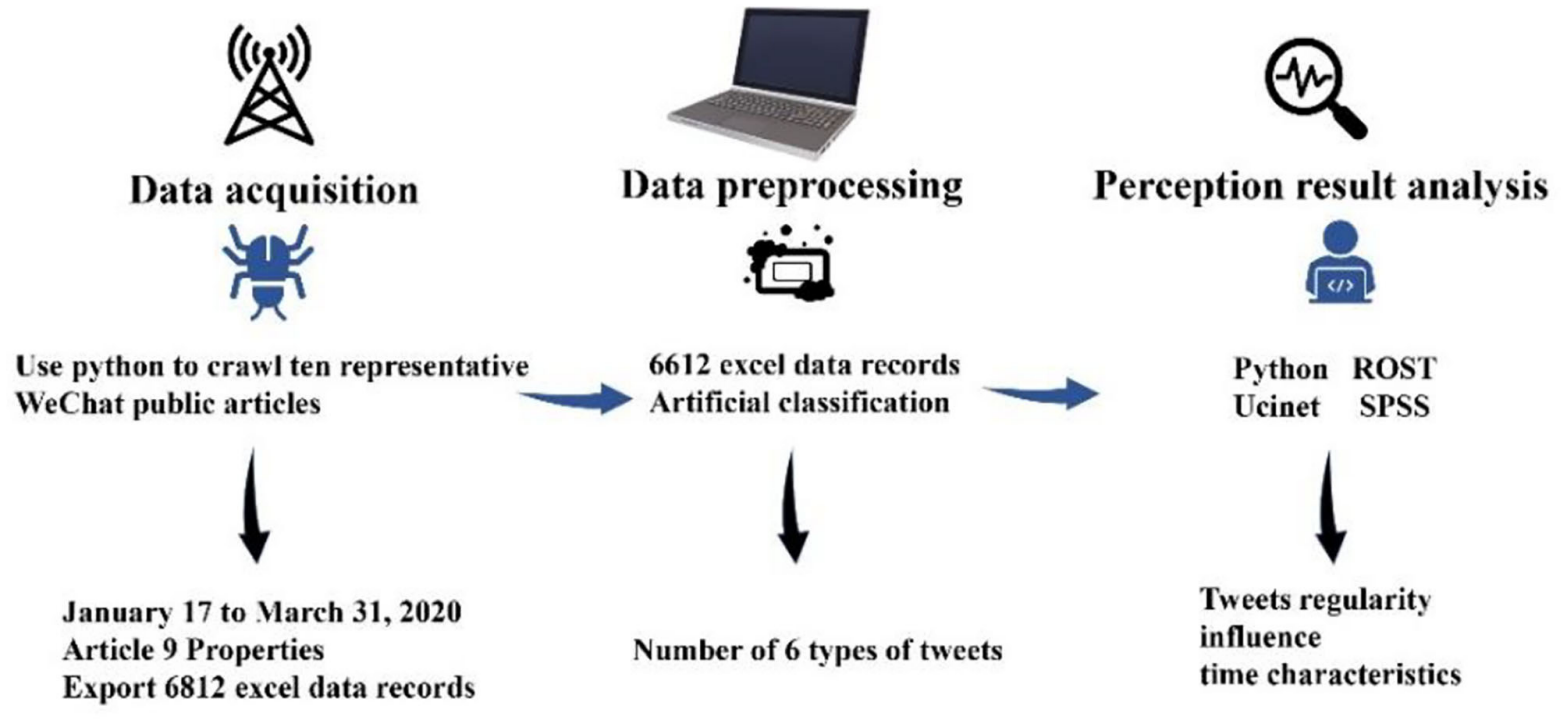
Research framework on the characteristics of WeChat official account tweets.

This study used Python, ROST, UCINET, and SPSS to analyze the 6,612 structured data collected in-depth. First, based on information metrics theory, we comprehensively analyzed tweets' regularity, influence, temporal characteristics, and other features. Second, the article titles were analyzed for co-occurrence according to the research methods related to information econometrics. To illustrate the results, we used the article titles high-frequency word co-occurrence map, WeChat public number independence strength map, and high-frequency word cloud map. Finally, in this COVID-19 pandemic, we summarize and analyze the characteristics related to the WeChat official account with strong communication power and credibility. This research involved four main steps: (1) Crawl the data of 9 attributes of 10 WeChat official accounts tweets using Python to form an Excel data summary table. (2) Use ROST to split the tweet title with high-frequency words to form a high-frequency words statistics table. (3) Visualize the data and analyze the patterns based on relevant statistics using Excel and SigmaPlot 14. (4) Use UCINET to visualize the independence strength and high-frequency word co-occurrence graphs of the WeChat official account. The resulting data were also validated using the SPSS tools (Figure 1).

## Results

### Time Characteristics of the Number of WeChat Official Account Articles

**Figure 2:**
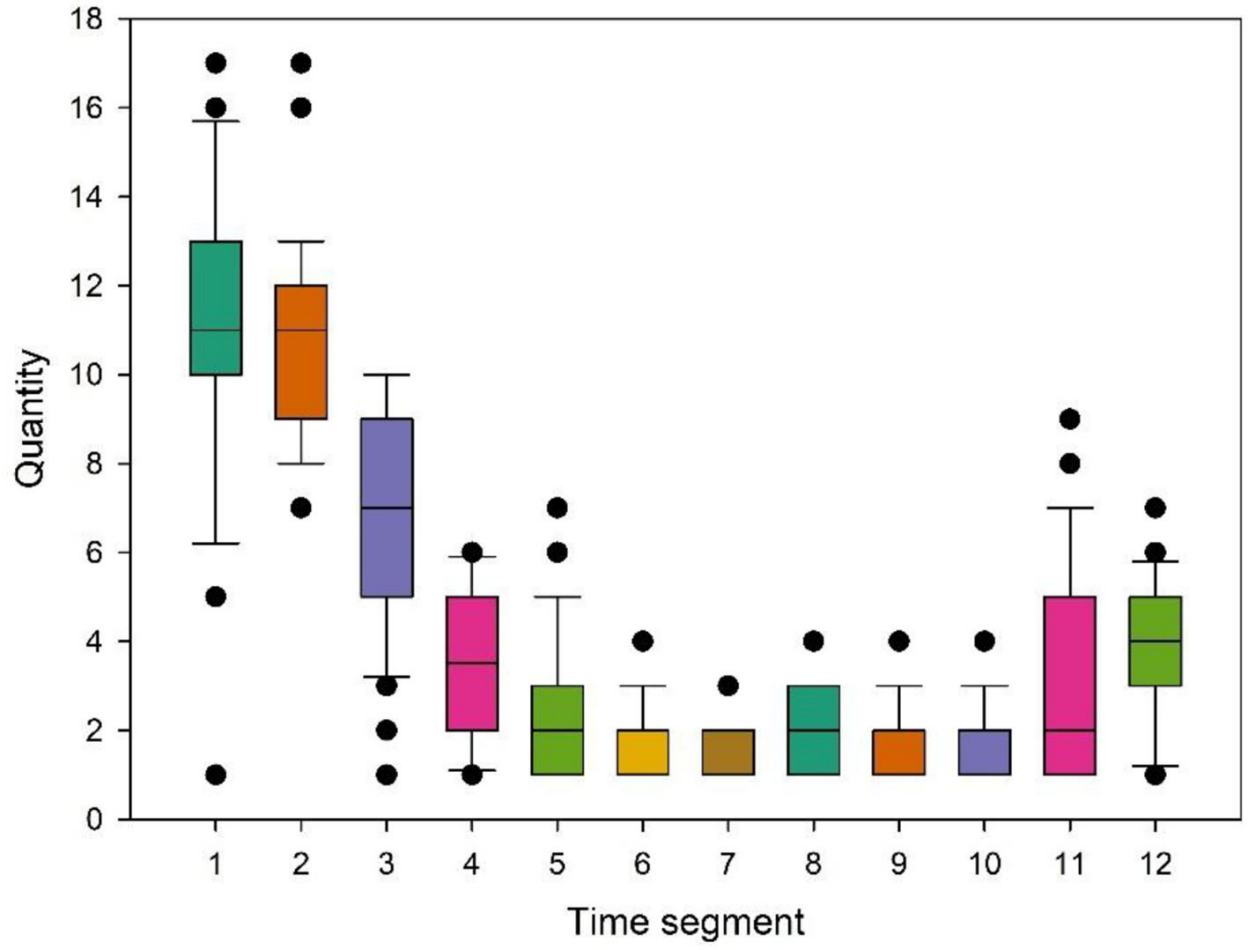
Box plot of the number of articles on WeChat official in each time segment.

This study first used python to collect daily article publication data of the Health Sichuan official microblogging public articles from January 21, 2020 to January 21, 2021, for 12 months. Then, a box diagram is used to intuitively judge the propagation law of the number of articles published on the official account over time (Figure 2). The number of articles quickly formed a small peak at the beginning of the outbreak and gradually leveled off over time, with the lowest point appearing within the seventh period and showing small fluctuations subsequently. At the same time, this study also counted the number of newly confirmed cases of COVID-19 from the Chinese government website and the Central People's Government's website during the same period. After Pearson correlation analysis (Table 4), it was found that the number of articles published by the WeChat official account and the number of newly confirmed cases (*r* = 0.721; *P* < 0.01) show a significant positive correlation. Therefore, this study concluded that the information on the WeChat official account articles could well reflect the information dissemination status of the COVID-19 pandemic (33).

**Table 4:** Pearson correlation coefficient.

**Variable**	**Number of articles**	**Newly confirmed cases**
Number of articles	1	0.721**
Newly confirmed cases	0.721**	1

**p <0.01.

**Figure 3:**
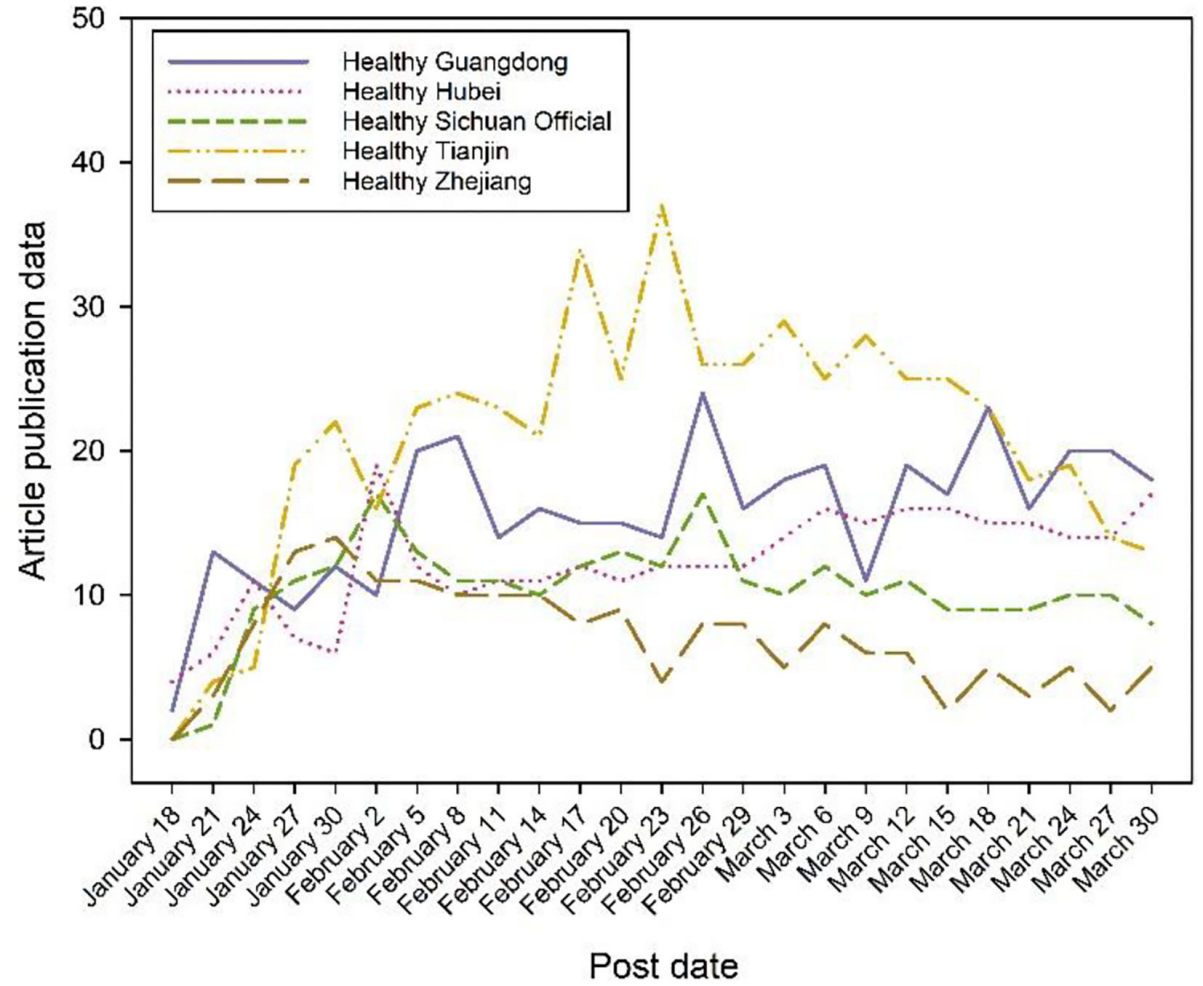
Daily trend of the number of tweets from different WeChat official accounts.

Next, the top five WeChat official accounts regarding the number of tweets were analyzed for volume time trends (Figure 3). The graph shows that Health Tianjin's tweets are significantly higher than the other four; the peak number of tweets is also earlier than the other public numbers, which indicates that Health Tianjin has some foresight and initiative to develop events. Health Zhejiang's tweets are at their lowest peak and have subsequently been on a downward trend. Health Zhejiang lacks observation of events compared to the other WeChat official accounts. In late January, after Zhong Nanshan proposed the phenomenon of human transmission, the number of tweets showed a small peak. The COVID-19 pandemic became increasingly heated in February, with the number of tweets reaching a mountain and a constant surge of outbreaks around the country. Public concern about confirmed diagnosis data and protective measures rose sharply, and the number of tweets on various types of information and rumors from the WeChat official account tweets remained high. The situation of the COVID-19 pandemic was effectively controlled in March, and the number of tweets gradually decreased.

**Figure 4:**
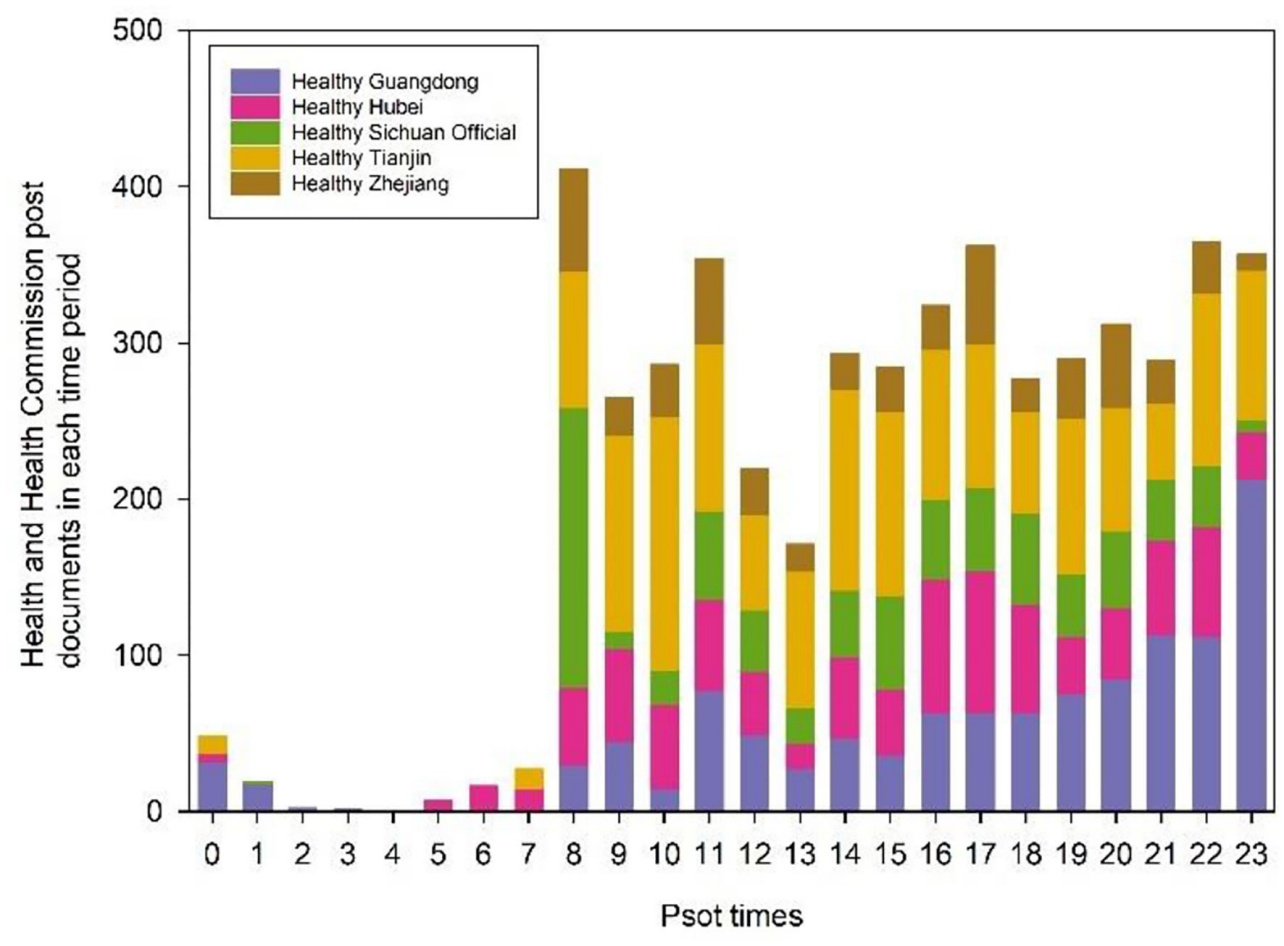
Trend of the number of tweets of different WeChat official accounts in each time period.

Next, we analyze the number of tweets from the top five WeChat official accounts for each time of the day (Figure 4). The overall change in the number of tweets over the day was a smooth progression starting at a low level, a sudden rise to a peak, followed by a decline, and then arising again in the afternoon. There are almost no tweets from 0 to 7 o'clock, which is the sleeping time of most people and is not conducive to news dissemination. 8:00 is the peak of the day for tweets, and Health Sichuan official mostly chooses to fix tweets at this time to let the news spread to the public first. Health Tianjin is always at a high level of tweets from 8:00 to 23:00, delivering first-hand news to the public. Healthy Hubei prefers to publish tweets between 16:00 and 17:00 to keep the audience updated after hours. Health Guangdong sets a fixed publishing time of 23:00, before people go to bed, to publish tweets, maximizing people's reading time before bedtime. Other public numbers tweeted time to cover almost the whole day, mainly because this event was watched by people worldwide, and WeChat official accounts of government media wanted to make sure the public got all the news in time.

### Analysis of Independence Among the WeChat Official Accounts

**Figure 5:**
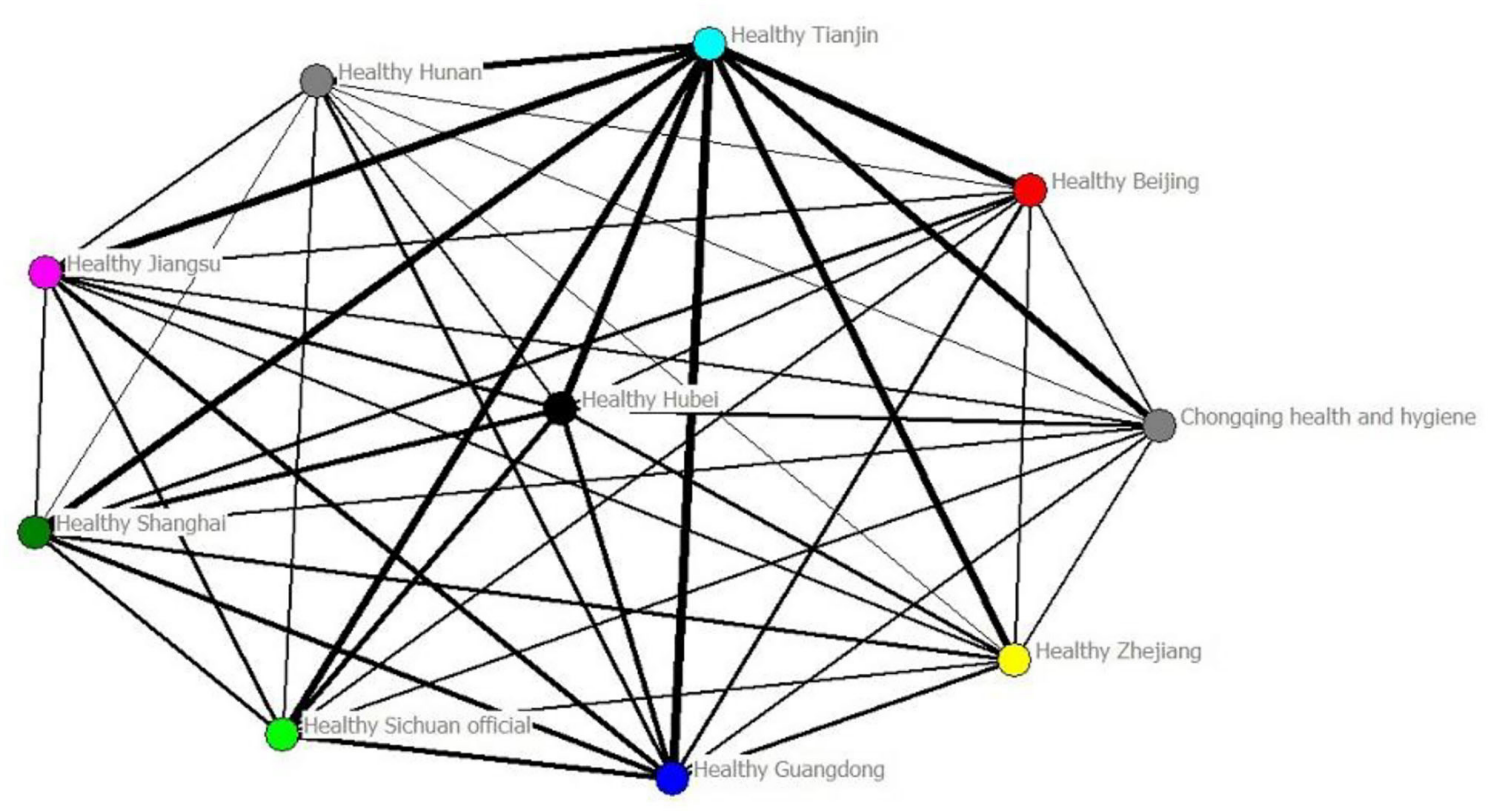
Similarity-based visualization mapping of WeChat official account independence networks.

The frequency of overlapping tweets among WeChat publics reflects the level of independence among WeChat publics (34). After processing the data, the independence matrix of 10 WeChat official accounts based on the number of repeated tweets was obtained by a self-coded algorithm. The independence matrix graph was obtained using the UCINET visual analysis tool (Figure 5). The number of samples in this treatment is small, there are no isolated nodes, and the results of this visualization reflect the similar relationship among the WeChat official accounts. The network density of the graph is 106. The dot in the graph represents the WeChat official account, and the bubble size represents the sum of the independent strength of this WeChat Public and the rest of the WeChat Public. The top three are Health Tianjin, 1,779 times; Health Guangdong, 1,136 times; and Health Hubei, 1,000 times. The thickness of the connecting line represents the strength of independence between the WeChat Public, and the independence strength between Health Tianjin and Health Guangdong is the strongest. Therefore, the similarity between Health Tianjin and Health Guangdong is the highest.

### Analysis of the Content of the WeChat Official Account Tweets

#### Distribution of Content Types of Tweets

This study counted the percentage distribution of each type of content in each WeChat official account (Table 5). The table shows that the overall percentage of science and disinformation-type tweets is the largest among all public numbers, and the overall percentage of negative impact is the smallest. Except for Health Beijing, the lowest percentage of content types tweeted by the remaining WeChat official account is negative impact. Meanwhile, health Beijing reported 66.12% of epidemic overview releases, significantly higher than other WeChat official accounts. In contrast, the percentage of tweet types addressing the epidemic impact was significantly less than other WeChat official accounts. Health Sichuan official is the only WeChat official account with the highest percentage of science and disinformation types, accounting for 32.17%.

**Table 5:** Percentage of each type of tweet in each public number.

	**Healthy Beijing**	**Healthy Guangdong**	**Healthy Hubei**	**Healthy Hunan**	**Healthy Jiangsu**	**Healthy Shanghai**	**Healthy Sichuan official**	**Healthy Tianjin**	**Healthy Zhejiang**	**Chongqing Health and hygiene**
**Negative Impact**	1.65%	1.53%	4.98%	1.43%	0.21%	0.00%	0.00%	0.25%	0.18%	0.00%
**Science and**	7.85%	22.91%	25.88%	5.71%	21.89%	40.00%	32.17%	31.08%	22.10%	21.36%
**disinformation**										
**Epidemic prevention**	17.36%	12.78%	26.99%	40.00%	16.21%	3.33%	24.64%	21.69%	25.41%	28.13%
**and control measures**										
**Epidemic overview**	66.12%	11.24%	15.60%	20.00%	16.63%	52.22%	10.64%	14.31%	13.81%	23.00%
**release**										
**Epidemic impact**	0.41%	3.24%	13.38%	8.57%	2.74%	1.39%	3.50%	1.01%	7.18%	1.85%
**Epidemic line**	6.61%	48.30%	13.16%	24.29%	42.32%	3.06%	29.05%	31.65%	31.31%	25.67%
**assistance stories**										

**Figure 6:**
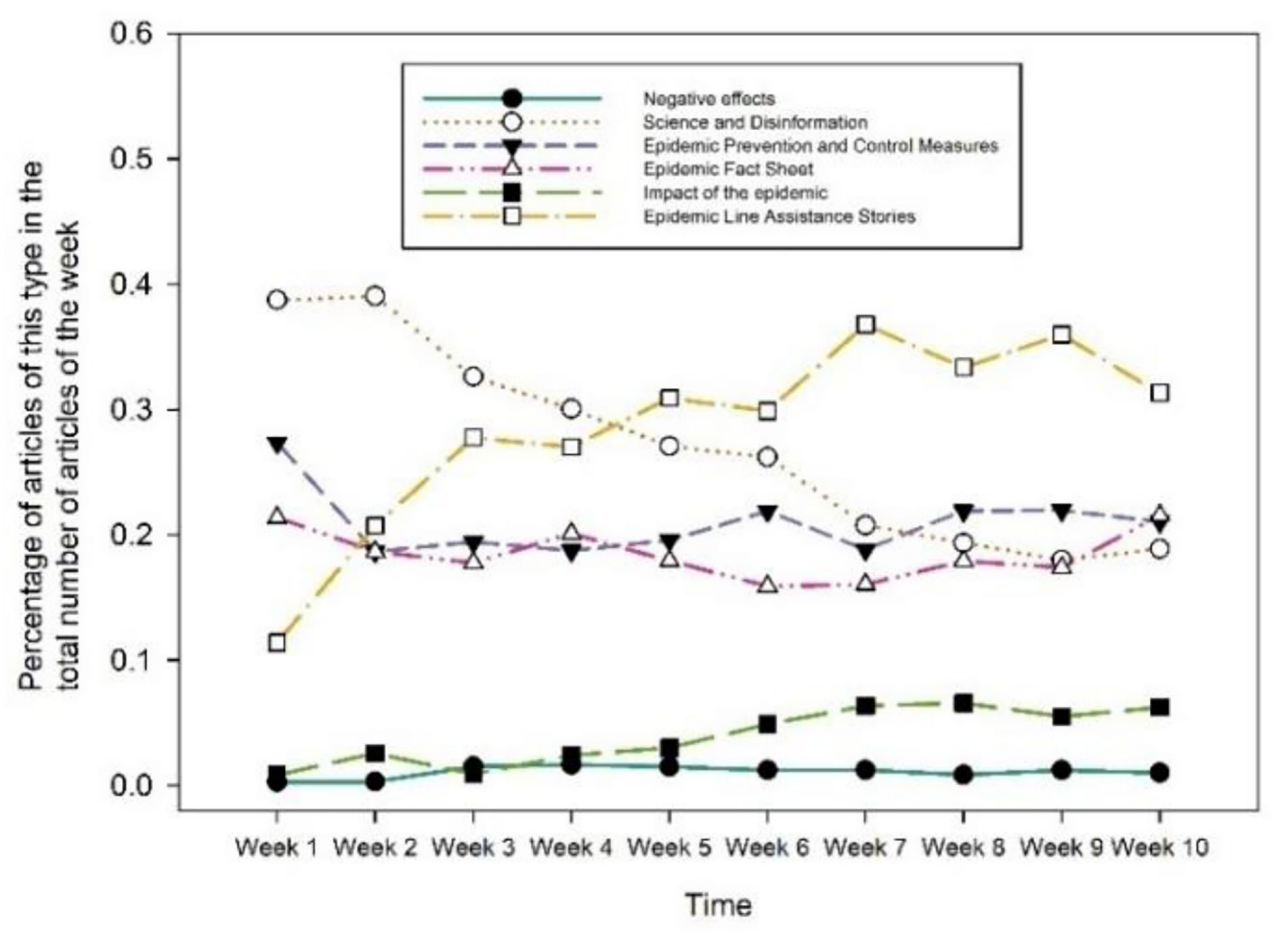
Tweets with posting time by content type.

This data collection was divided into 10 weeks, and the proportion of each type of article to the total number of tweets for that week was plotted (Figure 6). There are significant differences in each type of tweet variation over the collection time or even the exact opposite. The number of science and disinformation tweets shows a significant downward trend. In the first and second weeks, science and disinformation tweets accounted for the most significant percentage of all sorts of tweets, indicating that what the public most needs right now is the science of how to do it. The epidemic line assistance stories tweets showed a significant upward trend, accounting for a significantly higher percentage than other types from week 5 to week 10, when the epidemic situation was under control and the epidemic review gradually increased. Because of the rising content of resumption of work and school from week 6 onwards, the number of epidemic impact type tweets steadily grew. As the pandemic normalized by March, the percentage of tweets about epidemic preventive and control measures, epidemic overview releases, and negative impact stayed nearly unchanged.

#### Content Analysis of High Communication Power Tweets

The Single Tweets Communication Index (STCI) based on the enhanced WCI (WeChat Communication Index) established by Wang Lei et al. was utilized in this work to further extract the content of tweets with the high-propagation index for analysis (Formula 1), where *R* is the total number of tweets read from the date the tweet was posted to the data collection date, *Z* is the total number of tweet likes from the date the tweet was assigned to the data collection date, and *d* is the number of days included from the tweet's date to the data collection date. According to the STCI calculation based on the 6,612 tweets collected, 67 WeChat official account tweets with an STCI >800 were obtained. Some of the high-STCI tweets are shown in Table 6.


(1) $$\begin{array}{l} STCI\;=\;{\left\{60\%\;*\;\left(20\%\ln\left(\frac{R}{d}+1\right)+80\%\;*\;ln\left(R+1\right)\right)+40\;*\;\left(20\%\;*\;ln\left(10\;*\;\frac{Z}{d}+1\right)+80\%\;*\;ln\left(10\;*\;Z+1\right)\right)\;\right\}}^{2}\;*\;10 \end{array}$$

**Table 6:** High-STCI WeChat official account tweets' statistics (partial).

**Title**	**STCI**	**Public nickname**	**Type**
Public places without masks will be punished! In addition, Guangdong issued a strict epidemic prevention notice	1,090	Healthy Guangdong	2
Shock! From tonight, the whole city of Hangzhou bright screen to pay tribute to the soldiers in white!	1,090	Healthy Zhejiang	4
What are the symptoms of pneumonia in novel coronavirus infection? Here's everything you want to know!	1,035	Healthy Hubei	3
Twenty-five new cases in Guangdong! First confirmed cases were reported in Shantou and Dongguan	979	Healthy Guangdong	1
19 new cases in Guangdong! 1 case confirmed in Heyuan for the first time	976	Healthy Guangdong	1
Zhejiang actively prevents and controls the new coronavirus infection of pneumonia	951	Health Zhejiang	1
Huaxi Hospital was tightly guarded; out-of-town children's chaperones were found to be confirmed new crowns, close contacts, all isolated	946	Health Sichuan official	2
Pneumonia outbreak notification of novel coronavirus infection in Zhejiang Province	935	Health Zhejiang	1
The 48 h of entry of the new coronavirus into the body	924	Health Hubei	4
How long can coronavirus live in the air? How to protect yourself adequately? Take these three tips!	915	Health Hubei	1

The most WeChat official accounts publishing higher STCI tweets are Health Hubei, Health Guangdong, and Health Zhejiang, with 26, 16, and 15 tweets, respectively, according to the data of STCI higher than 800 tweets. Each type's tweets were 26, 17, 15, 7, 2, and 1, respectively. It can be seen that the content of high STCI tweets is mainly focused on epidemic overview release, epidemic prevention and control measures, science, and disinformation. The highest STCI was 1,090 for epidemic overview release and epidemic line assistance stories-type tweets issued by Health Guangdong and Health Zhejiang. The epidemic overview release of tweets presents the situation and changes of the COVID-19 pandemic with data, and these articles get the most attention from the public. The epidemic prevention and control measures type of tweets is closely related to people's lives and the economy, keeping the public informed of national policies and promptly controlling the spread of the epidemic. Finally, science and disinformation tweets functionally satisfy the public's desire for knowledge, enhance the public's sense of participation and security, and boost the public's positive mindset.

### Analysis of the Title of Government Media WeChat Public Tweets

#### High-Frequency Word Analysis

**Figure 7:**
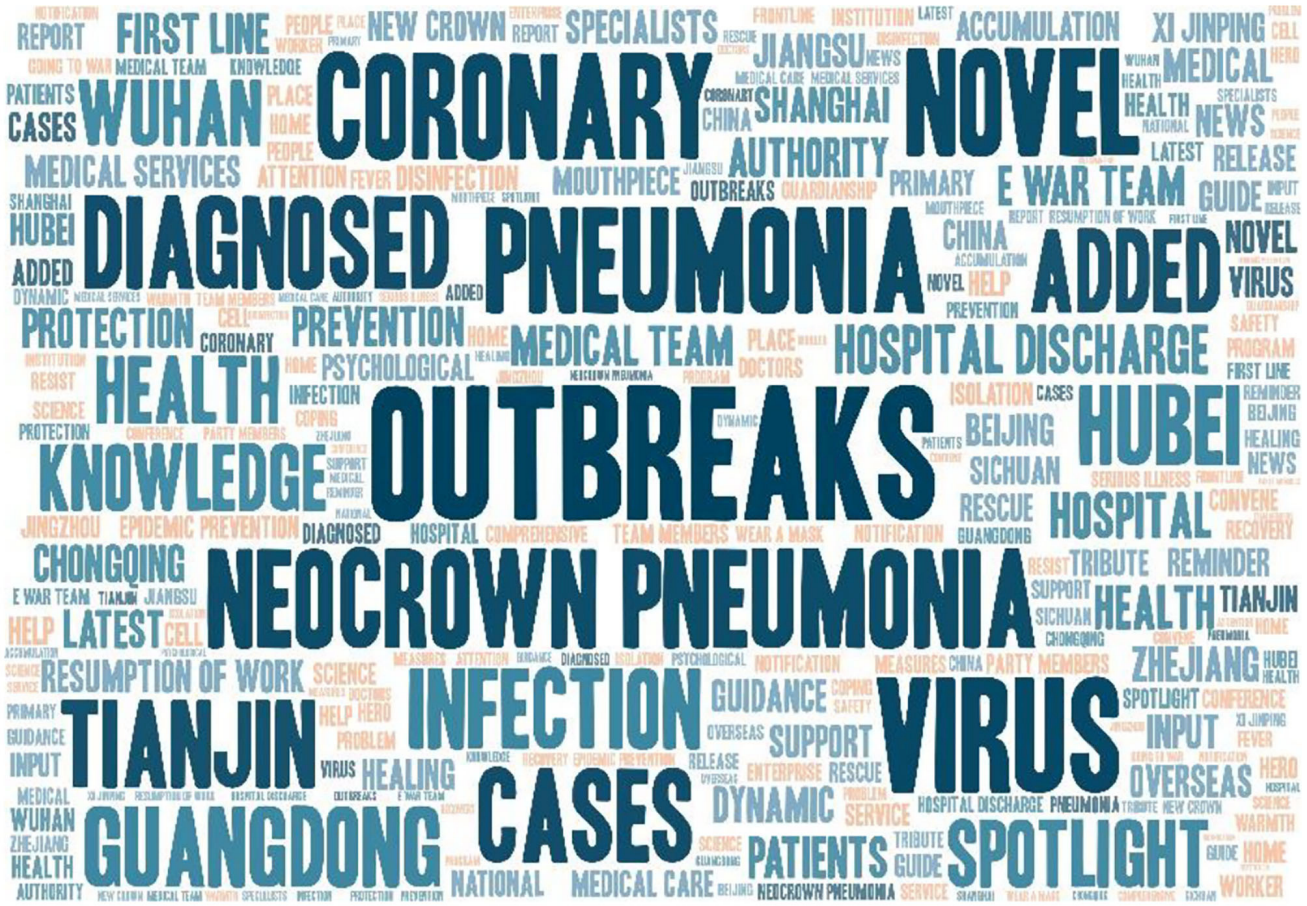
Tweets title high-frequency words word cloud map.

A total of 5,673 nouns, verbs, and gerunds with actual meaning were extracted from 6,612 tweet titles using the ROST tool for word separation. Then, using the BIBEXCEL tool, we counted the frequency of words and output them in order. After selecting words with a frequency greater than 60 times and removing words with no real meaning, we got a total of 100 high-frequency words for tweet titles (Table 7). The high-frequency word cloud was also mapped (Figure 7). The highest frequency word was for epidemics, which occurred 1,479 times. The 10 most frequent words closely followed by the public were outbreak, virus, novel, coronary, pneumonia, new coronary pneumonia, cases, diagnosed, Tianjin, and added, all of which were associated with the epidemic overview release.

**Table 7:** Tweets title high-frequency words (partial).

**Vocabulary**	**Word frequency**	**Vocabulary**	**Word frequency**	**Vocabulary**	**Word frequency**	**Vocabulary**	**Word frequency**
Outbreaks	1,479	New coronary pneumonia	806	Infection	540	Knowledge	369
Virus	1,089	Cases	723	Guangdong	536	Wuhan	361
Novel	949	Diagnosed	713	Hubei	527	Hospital Discharge	359
Coronary	923	Tianjin	635	Health	380	Hospital	328
Pneumonia	914	Added	577	Spotlight	380	Health	294

#### High-Frequency Words Coexist in the Network

A social network analysis of high-frequency words was used to create a matrix of 100 high-frequency words co-occurrence with a word frequency of more than 60 times in common in this study (35). To reduce the frequency gap, the high-frequency words co-occurrence matrix was transformed into a phase difference matrix by calculating the Ochiai coefficient through Formula 2. *y* is the Ochiai coefficient, *X*_*AB*_ is the frequency of *A* and *B* appearing together in a title twice, and *X*_*A*_ and *X*_*B*_ are the frequencies of *A* and *B* words, respectively.


(2) $$\begin{array}{l} y=\frac{X_{AB}}{\sqrt{X_{A}}\sqrt{X_{B}}} \end{array}$$

**Figure 8:**
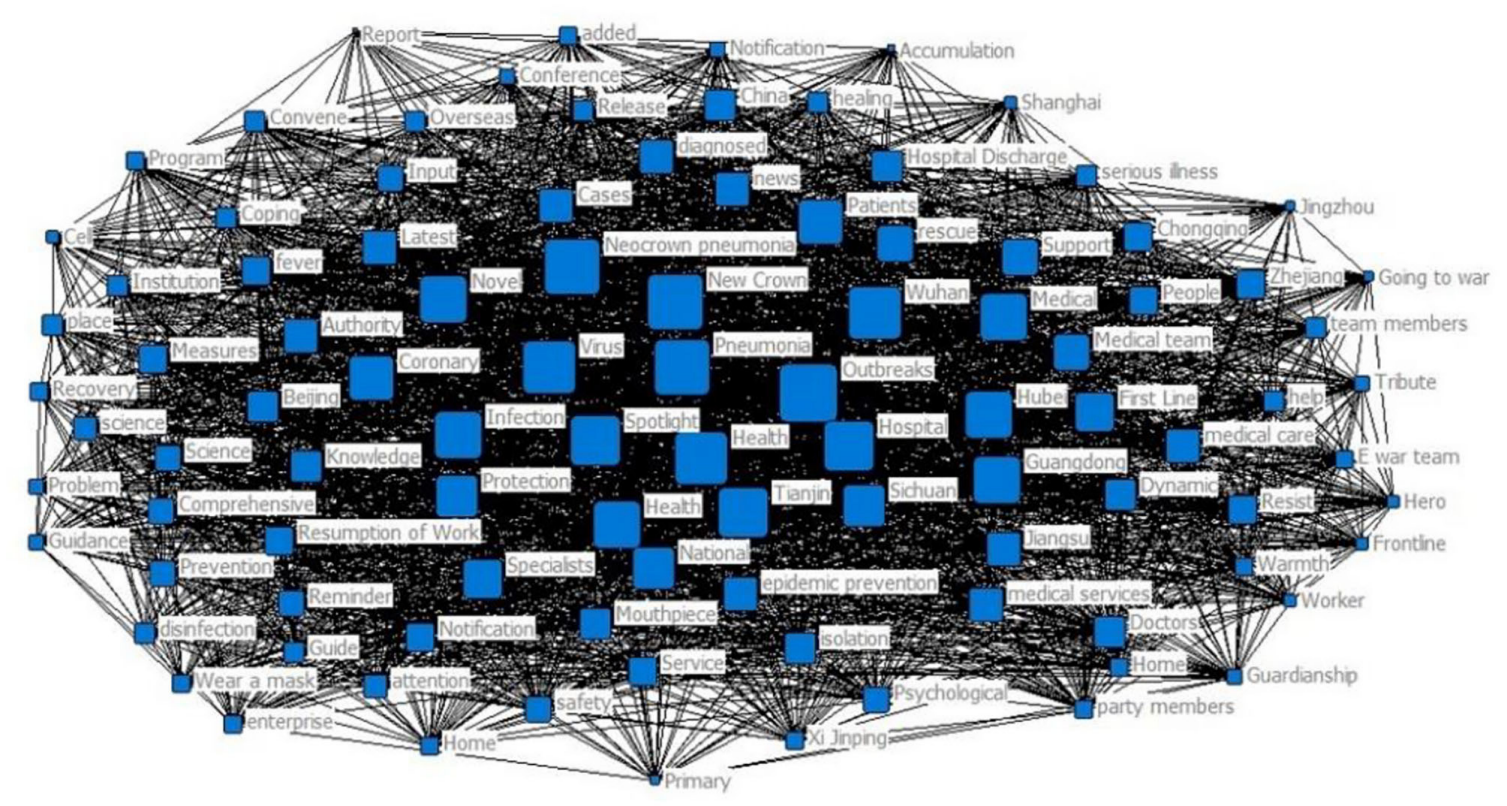
Tweets high-frequency words co-occurring network mapping.

The 100^*^100 high-frequency word co-occurrence was imported into the UCINET software to draw the high-frequency word co-occurrence network map (Figure 8). The overall network density of this network is 5.481. The graph shows that the wider the rectangle, the greater frequency of high-frequency word co-occurrence. Keywords with high point degree centrality include pneumonia, virus, novel, coronary, outbreak, case, and confirmed. It is consistent with the analysis results of high-frequency words of tweet titles, which occupy the core position of the title word co-occurrence network, are related to the science and disinformation and the epidemic overview release, and are the hot spots of tweets for this public health emergencies.

## Discussion

### Principal Findings

This study used a Python crawler, statistical analysis, text classification, and social network analysis tools to explore the characteristics of tweet information of the WeChat official account of the Chinese government and improve the information governance ability of the WeChat official account through the analysis of tweet information characteristics. It provides countermeasure support for the government to deploy public health emergencies from the time, content, and interactivity dimensions. This study has four key findings.

First, the daily number of articles tweeting information from the WeChat official accounts of the Chinese government showed a significant positive correlation with new confirmed cases of COVID-19 in China (*r* = 0.721, *P* < 0. 01). This finding suggests that the COVID-19 pandemic developed in WeChat in parallel with the new crown pneumonia outbreak in China. In line with this finding, there was a positive correlation between the daily number of posts related to the H7N9 epidemic on Weibo and the number of deaths from H7N9 avian influenza infection (36). The spread of public opinion information thus appears to be positively related to the spread of an epidemic virus. Accordingly, a positive correlation was found between the spread of information in WeChat official account tweets and the spread of the epidemic virus. Therefore, the distribution pattern of information tweeted by the WeChat official account can be used to determine the spreading status of an epidemic virus and assist the government in obtaining epidemic data on time.

Second, the WeChat official account with independent and innovative micro letter public numbers has stronger communication power. This is one of the significant findings of this study. The more repetitive the tweet content, the lower the probability of being reproduced and the lower the public's desire to spread it. Health Zhejiang published the most liked articles 18,372 times. Health Zhejiang is more independent and has more innovative material than other public numbers. Health Zhejiang has published 1,168 pieces of original content from creating the WeChat public until now, the highest among all WeChat public. Most of its high reprint rate articles are original articles. The government should adopt various means to enhance the visibility of the WeChat official account, such as printing leaflets, establishing publicity windows, and improving interactive services and information dissemination services, to enhance the public's experience. By enhancing their independence and innovativeness, WeChat official accounts continue to strengthen their public communication capabilities, enhance public credibility, and lay the foundation for the government to effectively control the spread of public opinion.

Third, the key factors affecting the power of communication are the tweets' value, interest, and moving nature. The value is reflected in the tweets with information on epidemic data notification, epidemic policy notification, epidemic prevention and control means, and negative news views, allowing the public to grasp the situation and helping the public establish the correct values quickly. Interesting is reflected in titles like “48 h of COVID-19 Entering the Body” and “The Last Strange Death of the New Coronavirus,” which are interesting, innovative, and rapidly capture the public's attention. Moved is reflected in titles like “shock! Tonight, the whole city of Hangzhou bright screen to pay tribute to the soldiers in white!” and “61 pictures, although a bit too many, but each one makes people want to cry!”, to meet the sense of public participation, so that the public to produce emotional touch. Brightening the title name and enhancing the quality of tweets is the primary means to attract users' attention to the WeChat official account (37), as special article titles are more attractive to users (38). According to this feature, when publishing content related to the epidemic, the WeChat official account needs to enhance the headline's value, interest, and moving nature, improve the dissemination and readability of information, control the direction of public opinion, and guide the masses promptly.

Fourth, the level of activity of government media public plays a vital role in improving credibility. The percentage of Health Hubei's tweets viewed more than 10,000 times is 0.86, and the number of articles it has published is among the highest in the WeChat public. Health Hubei's articles have more likes than other WeChat official accounts because they have a fair coverage of content and comprehensive coverage. Among the different homogeneous WeChat official accounts, the public prefers active WeChat official accounts to follow. The richer the content, the more the public can gain a sense of identity, thus enhancing the credibility of the WeChat official accounts in the public's mind. Government media should improve news acumen, capture the latest information, meet the public's right to know and bring the correct general measures to prevent and treat public health emergencies.

### Strength and Limitation

#### Theoretical Significance

Under the information epidemic, the spread of information opinion on social media can strongly influence people's behavior and change the government's decision deployment (39). This study found that changes in the number of articles published by WeChat, changes in content, and the progress of the COVID-19 pandemic are nearly synchronized. (1) Find a way to govern the communication power of the WeChat official account; the first is to enhance independent innovation that can effectively improve the communication power of the WeChat official account, and the second is to enhance the value, interest, and moving of tweets that also has a strong effect on the communication power of WeChat official account. (2) This study discovered the factors that influence WeChat official accounts' credibility improvement: first, activeness has a strong positive effect on WeChat official accounts' credibility improvement, and second, independent innovation ability also has a positive effect on credibility improvement. These findings provide a theoretical basis for the WeChat official account to guide and control the development of public opinion and enhance communication power and credibility in the face of public health emergencies.

#### Practical Significance

This research can help the Chinese government develop public opinion information dissemination management systems and public guiding strategies in a public health emergency. First, through the information characteristics of the articles released by the WeChat official account, the government and related departments can improve the communication power of their WeChat official accounts, guarding the leading position of public opinion and enhancing the credibility of the government's WeChat official accounts while grasping the direction of public opinion. For example, graphic or video interpretation enhances the moving nature of the public, triggers empathy, and guides the masses to the correct values. In addition, the government should pay attention to the impact of environmental changes on the spread of major public health emergencies and make an excellent early warning mechanism for public opinion dissemination and epidemic prevention and control. Second, this study provides a basis for establishing an appraisal mechanism for the operation of WeChat official accounts and a quantitative assessment of the quality and effectiveness of the promotion of WeChat official accounts. Finally, this study provides methods for the WeChat official account to improve its operation and management and strengthen its ability to handle public opinion on public health emergencies.

This study has some limitations. First, this research focused on the WeChat official account platform in China. Therefore, the conclusion may not apply to other social media platforms in other countries (e.g., Twitter). Second, the issue of the number of people in each province and the ratio of people using the Internet was not considered. Third, the main research topic is the WeChat official account of the government, and there is a lack of research on the WeChat public the private media. In addition, the research on other propaganda platforms of government media, such as Tiktok, needs to be further deepened, and these issues will be addressed in future research.

## Conclusion

This study analyzed the tweet information of 10 WeChat official accounts of the Chinese government and obtained the following conclusions. First, the number of articles and content changes on the WeChat official account are consistent with the development of the COVOD-19 emergency, which can well reflect the progress of public health emergencies and allow the government to control the critical nodes of public health emergencies through the content and number of articles. Second, the more valuable, interesting, and moving the articles published by the WeChat official account are, the stronger the spreading power of its articles. According to the characteristics of high STCI tweets, the WeChat official account should take measures such as brightening the titles of tweets, enhancing the attractiveness of the content of tweets, improving the communication power of tweets, and guiding the direction of public opinion. Finally, the WeChat official account with stronger independent innovation and higher activity has higher credibility. When the WeChat official account of the government understands the features of public health crises and produces unique pieces based on those qualities, the public's trust in the government will be enhanced, allowing the government to take the lead in public opinion and avert additional epidemics.

## Data Availability Statement

The raw data supporting the conclusions of this article will be made available by the authors, without undue reservation.

## Author Contributions

Conceptualization, methodology and visualization: MW and ZL. Software: MW and JZ. Investigation: MW, YR, and ZL. Data curation: JZ and YR. Writing—original draft preparation: MW. Writing—review and editing: ZL. All authors have read and agreed to the published version of the manuscript.

## Funding

This research was funded by the Major Projects of Sichuan Province in the 13th Five-Year Plan for Social Sciences in 2020 (grant number SC20YJ002).

## Conflict of Interest

The authors declare that the research was conducted in the absence of any commercial or financial relationships that could be construed as a potential conflictof interest.

## Publisher's Note

All claims expressed in this article are solely those of the authors and do not necessarily represent those of their affiliated organizations, or those of the publisher, the editors and the reviewers. Any product that may be evaluated in this article, or claim that may be made by its manufacturer, is not guaranteed or endorsed by the publisher.
